# Situational judgment test validity: an exploratory model of the participant response process using cognitive and think-aloud interviews

**DOI:** 10.1186/s12909-020-02410-z

**Published:** 2020-12-14

**Authors:** Michael D. Wolcott, Nikki G. Lobczowski, Jacqueline M. Zeeman, Jacqueline E. McLaughlin

**Affiliations:** 1 The University of North Carolina Eshelman School of Pharmacy, 321 Beard Hall, Chapel Hill, NC 27599 USA; 2 The University of North Carolina Adams School of Dentistry, Chapel Hill, NC USA; 3 The University of North Carolina School of Education, Chapel Hill, NC USA; 4 Carnegie Mellon University, Pittsburgh, PA USA

**Keywords:** Cognitive interview, Empathy, Qualitative methodology, Response process, Situational judgment test, Think-aloud protocol, Validity

## Abstract

**Background:**

Situational judgment tests (SJTs) are used in health sciences education to measure examinee knowledge using case-based scenarios. Despite their popularity, there is a significant gap in the validity research on the response process that demonstrates how SJTs measure their intended constructs. A model of SJT response processes has been proposed in the literature by Robert Ployhart; however, few studies have explored and expanded the factors. The purpose of this study was to describe the factors involved in cognitive processes that examinees use as they respond to SJT items in a health professions education context.

**Methods:**

Thirty participants—15 student pharmacists and 15 practicing pharmacists—completed a 12-item SJT designed to measure empathy. Each participant engaged in a think-aloud interview while completing the SJT, followed by a cognitive interview probing their decision-making processes. Interviews were transcribed and independently coded by three researchers to identify salient factors that contributed to response processes.

**Results:**

The findings suggest SJT response processes include all four stages (comprehension, retrieval, judgment, and response selection) as initially proposed by Ployhart. The study showed factors from other published research were present, including job-specific knowledge and experiences, emotional intelligence, and test-taking. The study also identified new factors not yet described, including identifying a task objective in the scenario, assumptions about the scenario, perceptions about the scenario, and the setting of the item.

**Conclusions:**

This study provides additional SJT validity evidence by exploring participants’ response processes through cognitive and think-aloud interviews. It also confirmed the four-stage model previously described by Ployhart and identified new factors that may influence SJT response processes. This study contributes to the literature with an expanded SJT response process model in a health professions education context and offers an approach to evaluate SJT response processes in the future.

**Supplementary Information:**

The online version contains supplementary material available at 10.1186/s12909-020-02410-z.

## Background

Situational judgment tests (SJT) have attracted substantial interest in health sciences education as an assessment methodology [1, 2]. The purpose of an SJT is to evaluate how an examinee would respond to scenarios commonly encountered in practice [3, 4]. During an SJT, the examinee reviews a hypothetical scenario and rates the effectiveness of potential responses to that scenario. SJT items measure examinee knowledge by identifying the response they believe is most appropriate to fulfill the job’s expectations—these expectations often coincide with the desired constructs measured [5]. Participants are then assigned a score based on how well their selections align with a key, frequently established using subject matter experts [6].

SJTs appear in admissions processes, capstones, and longitudinal assessments across various disciplines, including medicine, pharmacy, and nursing [2, 7–10]. Despite increasing popularity, interest in SJTs initially eclipsed research on the methodology as an assessment strategy [11]. Specifically, there were few attempts to establish validity evidence that distinguished what constructs were assessed and the elements involved in response processes [12]. It is imperative that assessments have sufficient validity evidence to support their interpretation and use [13].

Of the five sources of validity evidence recommended by the *Standards for Educational and Psychological Testing*, research on the response process during SJTs is a neglected area of research [12, 14–18]. At the time of this research, only two studies have investigated select components of the response process, and both included participants outside the health professions. One study characterized participant utterances to see alignment with the construct of interest, while the other focused on test-taking strategies [19, 20]. The absence of research restricts our understanding of the cognitive processes involved in answering SJT items.

The response process during any assessment or instrument includes the moment-to-moment steps required to think and make decisions [21]. Cognitive response processes include how data is accessed, represented, revised, acquired, and stored to address a question. The decision-making process then includes manipulating information in a series of steps influenced by existing knowledge, previous experience, or similar applications. In general, cognitive response processes associated with schema are considered domain-specific and may change depending on the setting [21].

When assessing response processes during assessments, validity evidence must demonstrate that test takers use cognitive processes in a coordinated fashion consistent with the theoretical and empirical expectations [22]. Evaluating the cognitive response process is often elaborate and varies based on the context or tasks assessed. Investigating cognitive response processes often includes think-aloud procedures and cognitive interviews conducted during a cognitive task analysis to create verbal reports for analysis [23, 24].

**Fig. 1:**
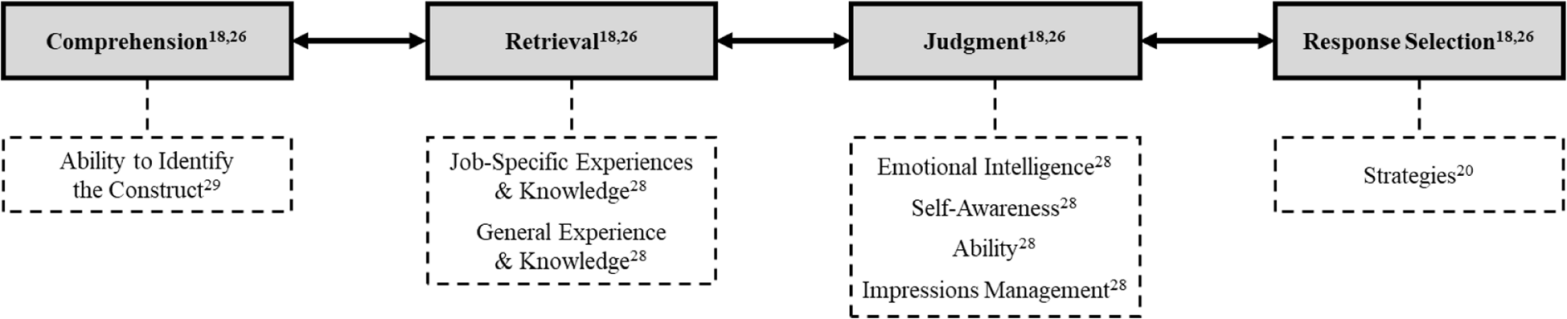
Adaptation of the SJT response process model based on Ployhart and additional research [18, 20, 26, 28, 29]

Ployhart proposed an SJT response model built on an existing four-stage framework originally produced by Tourangeau and colleagues describing the cognitive response process during surveys [18, 25, 26]. The model includes: (1) comprehension, (2) retrieval, (3) judgment, and (4) response selection [26]. During comprehension, the examinee reads, interprets, and understands the purpose of the question. Next, during retrieval, the examinee accesses long-term memories and knowledge relevant to the scenario. The examinee forms a judgment based on an integration of memories, knowledge, experiences, and other antecedents [27]. Finally, the examinee selects a response that is most consistent with their judgments. Ployhart also noted all stages of the response process could be influenced by other sources of construct-irrelevant variance (e.g., language barriers, interpretation issues, and impression management) and test-taker motivation [18]. Fig. 1 depicts a rendering of Ployhart’s existing model plus the additional factors identified from other research [18, 20, 26, 28, 29].

The purpose of this study was to identify salient factors of SJT response processes, thus addressing an important gap in the SJT validity evidence literature. This study focused on response processes during an SJT measuring empathy, an important construct in health professions education. This research provides a prototype for exploring and describing SJT response processes by addressing the question: *What factors are involved in cognitive processes when examinees respond to SJT items?* The research question was exploratory and aimed at building on the current understanding of SJT response processes while expanding to a health professions education context.

## Methods

### Participants

The study used a convenience sample of 15 student pharmacists enrolled in a Doctor of Pharmacy (i.e., PharmD) degree program and 15 practicing pharmacists with at least 5 years of experience. The sample size was deemed sufficient based on prior SJT response process research that showed saturation at smaller sample sizes [19]. In addition, the exploratory nature and the necessity to conduct in-depth interviews with participants made a smaller sample size more feasible and efficient. Participants received an alphanumeric identifier: students have an “S” label, and pharmacists have a “P” label with a number from one to 15. The University of North Carolina Institutional Review Board approved this study.

### SJT development

The research team created a new SJT to evaluate empathy (i.e., the construct of interest) given its multifaceted nature and relevance to healthcare [30, 31]. Empathy is considered a multidimensional construct that includes at least two factors: cognitive empathy and affective empathy [32–35]. *Cognitive empathy* refers to an individual’s ability to understand another person’s perspective versus being self-oriented [36]. This cognitive perspective includes being able to imagine alternative realities, to judge the difficulty of scenarios, and to “step into another person’s shoes and to step back as easily into one’s shoes again when needed.” [33] The other element, *affective empathy,* pertains to an individual’s ability to understand and internalize the feelings of others [37]. Also called emotional empathy, affective empathy relates to recognizing an individual’s emotional response and through their interactions with others [33].

Lievens’ construct-driven approach informed the design of SJT items for this study, incorporating theoretical and empirical evidence to inform sound instrument design [38]. Each item targeted one of the two empathy components (i.e., affective or cognitive empathy), so the overall score on the SJT was representative of the unidimensional construct of empathy. SJT items used a knowledge-based format (i.e., *should* do), as this format has evidence that it requires more job-specific and general knowledge [39, 40]. All items used ranking-response formats, as this required participants to analyze and discriminate among all options for each test item [41, 42]. To allow the participants ample time to answer each question, their response time was not restricted; however, the team anticipated participants would require at least 2 min per question.

The SJT design process followed a similar approach described in existing research and based upon literature from SJT design experts [10, 41, 42]. The first phase included a panel of subject matter experts (i.e., practicing pharmacists) who created 24 items evaluated by a second panel on three criteria: how well the item measured empathy, which empathy component was measured, and the perceived setting of the item. The final SJT included 12 items with a high level of agreement on the selection criteria. There were six items per empathy component (i.e., affective and cognitive empathy), with three items per component targeting job-specific knowledge (i.e., a healthcare setting) and three items targeting general domain knowledge (i.e., a non-healthcare setting). Table 1 includes a sample item and an item summary with a visual item map available in the supplemental appendix.

**Table 1:** Summary of the empathy SJT item content

Item label	Component	Setting	Item summary
CH1	Cognitive	Healthcare	A patient complains that the doctor never listens to them
CH2	Cognitive	Healthcare	A provider has trouble getting a medication history from a pharmacist
CH3	Cognitive	Healthcare	You suspect a patient is lying about their diabetes management
CN1	Cognitive	Non-healthcare	A friend is going to use unprescribed medications to help them study
CN2	Cognitive	Non-healthcare	A woman asks you to cut in line at a store when you’re late
CN3	Cognitive	Non-healthcare	Your family questions your sibling’s relationship status
AH1	Affective	Healthcare	A patient discusses the recent loss of a loved one
AH2	Affective	Healthcare	A nurse asks you to discuss a medication error with family
AH3	Affective	Healthcare	A family gets upset while you review their chemotherapy
AN1	Affective	Non-healthcare	A parent quickly becomes upset at a grocery store
AN2	Affective	Non-healthcare	A relative is upset about difficulty conceiving
AN3	Affective	Non-healthcare	A best friend is visiting and planning to drop out of college
**ITEM: CN2** You go to the store to pick up a few things you forgot for a presentation. While standing in line at checkout, someone approaches you and asks if they can cut in front of you. However, there are already 5 people behind you. They mention that their children are at home sick and they are trying to get back as quickly as possible. Letting the person go in front of you will definitely make you late for your presentation. *Rank each of the following response options based on how you* SHOULD *respond to the scenario. Use 1 to indicate the MOST appropriate response and 5 to indicate the LEAST appropriate response. There can be no ties or duplicates.* _____ Ask the people behind you if they would mind having the person go in front of you. _____ Acknowledge their situation and let them go in front of you. _____ Tell them no and that they need to get in line like all the others. _____ Ask the person what is wrong with their child and determine whether they cut can based on their response. _____ Tell them that you are also in a rush and ask if they could cut in front of the person behind you.			

Notes: *A* Affective Empathy, *C* Cognitive Empathy, *H* Healthcare Setting, *N* Non-Healthcare Setting; 1, 2, 3 = Item Number

### Data collection procedures

Recruited students and pharmacists participated in the study during May 2019; emails were sent through student and practitioner listservs managed by the University of North Carolina Eshelman School of Pharmacy. Students who participated had an opportunity to win a $25 Amazon® gift card while pharmacists were not offered an incentive for participating. Study participants met with the lead researcher (MW) for a 90-min one-on-one interview, including written consent, the think-aloud interview, the cognitive interview, and a written demographic survey. The interview protocols are available in the supplemental appendix.

During the think-aloud interview, participants completed the full 12-item SJT one item at a time. They were not allowed to revisit prior questions once they had finished. The item order was randomized for each participant to minimize order effects. Participants verbalized their thoughts as they completed the SJT during the think-aloud interview. The interviewer only intervened by stating, “keep talking” after periods of silence longer than 5 s [23]. The researcher did not ask participants to elaborate and describe their approach to limit introducing bias [23, 24, 43].

Following the think-aloud, participants completed the cognitive interview, where they were asked about their understanding of and approach to select SJT items. The difference between the think-aloud and cognitive interview is that the latter included questions about how participants solved each problem and why they made individual selection decisions. Participants had the opportunity to review each item and their responses as they answered the cognitive interview questions. However, participants could not change their submitted responses. The cognitive interview protocol organized questions to explore the factors relevant in their decision-making process, including those related to Ployhart’s model [18].

Due to time constraints, each participant answered questions about their responses for eight of the 12 SJT items. SJT items were evenly distributed among participants based on the empathy component assessed and the setting. In other words, participants completed four items from a healthcare setting, four items from a non-healthcare setting, four items measuring cognitive empathy, and four items measuring affective empathy. For each SJT item, there were a total of 20 cognitive interviews, including ten interviews with students and ten interviews with pharmacists.

SJT data and demographic survey responses were compiled into an electronic database (i.e., Stata®) and labeled using the unique participant identifier. Audio files from the interviews were converted to written transcripts using an online transcription service (i.e., Rev.com); transcripts were uploaded to qualitative analysis software (i.e., MAXQDA®). For the think-aloud interviews, the entire interview was maintained in its original composition and grouped by the participant type (i.e., student or pharmacist). For the cognitive interviews, segments of interviews were grouped according to the test item. For example, all cognitive interview questions related to item CH1 were grouped into one transcript for analysis and subdivided based on whether it was a student or a pharmacist to optimize data analyses.

### Data preparation & analysis procedures

Ployhart’s SJT response process model informed the initial codebook design for the cognitive interview analysis [18, 26, 28, 29]. Researchers were also permitted to inductively code text segments as “other” if they identified what they perceived to be an emerging code. The final codebook is available in the supplemental appendix. The coding process for the cognitive interview included a calibration phase followed by three rounds of coding conducted independently by two researchers. During the calibration phase, the researchers used a mock transcript from the pilot test of four SJT items. The two researchers independently coded the transcript according to the initial codebook and met to review discrepancies, generate example quotes for the codebook, and modify the codebook definitions as needed. The goal of the calibration phase was to allow the raters an opportunity to align coding expectations and resolve concerns before the official coding process [44].

After calibration, the cognitive interview coding included a step-wise approach commonly used in qualitative analysis of large data sets where two researchers are not required to code all data elements [44]. First, two researchers (MW and NL) independently coded approximately 30% of the transcripts (i.e., transcripts related to four SJT items). The researchers met to review the rater agreement, resolve discrepancies, and modify the codebook when necessary. The consensus is that a rater agreement above 80% indicates high consistency to permit a streamlined approach [44, 45]. The agreement for the first round was 80.2%; therefore, only one researcher (MW) independently coded another 30% of the transcripts in the next round. The second researcher (NL) then audited the coding process results from round two, and the two researchers met to resolve discrepancies. The second round had 97.7% agreement, so the lead researcher (MW) completed the final session coding with no audit. Coding of think-aloud interviews used the same process, with JZ served as the second researcher. During think-aloud interview coding, no new codes were added to the codebook. Rater agreement for think-aloud coding was 87.5% during the first phase (coding by both researchers) and 94.9% during the second phase (auditing by the second researcher).

**Fig. 2:**
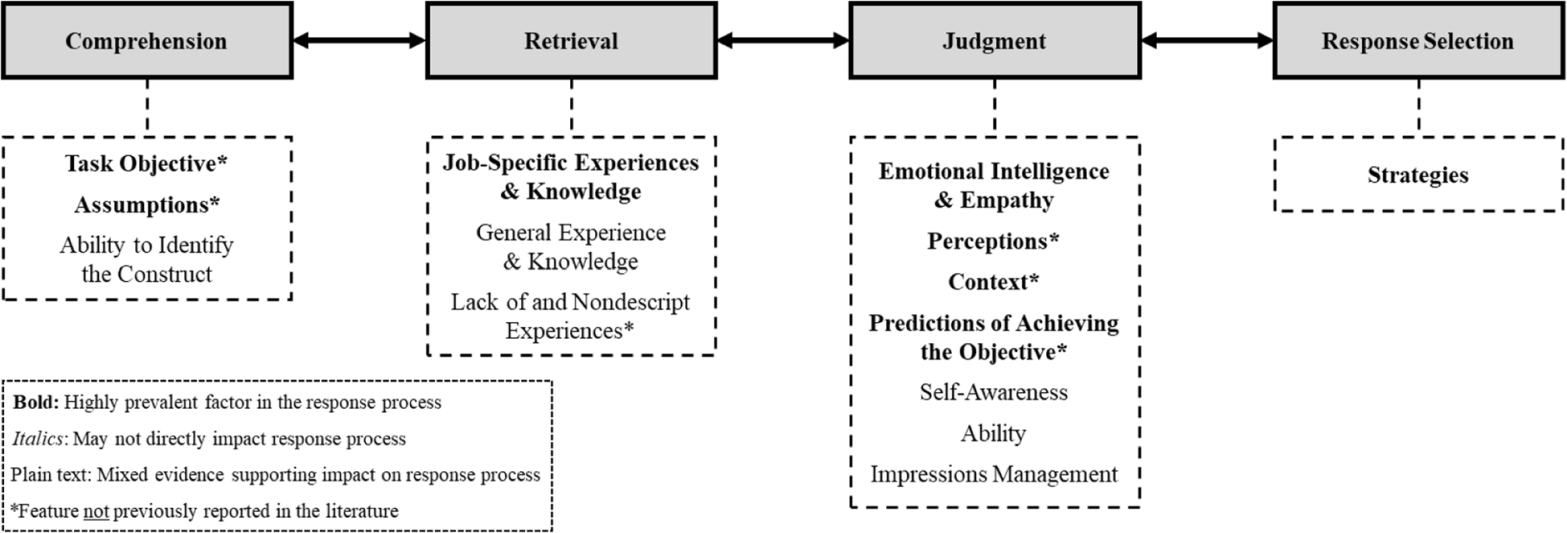
A revised model of SJT response processes based on the findings from this study

The team examined the prevalence and context of participant utterances from coded transcripts to identify patterns and relationships among the codes. There was evidence to support an underlying SJT response process from salient observations in the cognitive and think-aloud interviews. Thus, these findings supported the generation of a new SJT response process model (Fig. 2) [18, 26, 28, 29]. The supplemental appendix also includes a summary of SJT psychometric qualities and SJT results; a more detailed description is available elsewhere [46]. Overall, the findings suggest the SJT provided sufficiently reliable and valid data regarding empathy. Quantitative analyses of data are not presented in this paper as the focus was on the exploratory qualitative research related to SJT response processes. Of note, we did not conduct group comparisons using the qualitative data due to the small sample sizes and exploratory research aim—the focus was on generating a broad model to be tested later using quantitative methods.

## Results

### Participant characteristics

The student participants were predominantly female (*n* = 11, 73.3%) with a median age of 24 years (range 22–45 years). Most students were entering their third or fourth year of pharmacy school (*n* = 11, 73%), meaning they had experience working in a pharmacy practice setting through required clinical experiences. In addition, 13 students (87%) indicated working in a healthcare-related field outside of their coursework. Eight students (53%) reported working in a non-healthcare human services field with 1 year of experience being the median (range 0–10 years). Eighty percent (*n* = 12) of students reported they completed training about empathy; they most often cited coursework or classroom discussions regarding mental health and working with patients.

The pharmacists were predominantly female (*n* = 13, 86.6%) with a median age of 36 (range 29–51 years). All pharmacists worked in a university hospital setting across various practice disciplines, and they had a median of 8 years of experience as a licensed pharmacist (range 6–23 years). Most pharmacists completed residency training (*n* = 13, 87%) and were board-certified (*n* = 11, 73%), indicating these individuals have extensive training in specialty areas and providing advanced patient care. Eleven pharmacists (73%) reported previously working in a non-healthcare human services field with a median of 4 years of experience (range 0–10 years) outside of pharmacy. Only 33% (*n* = 5) of pharmacists reported having training about empathy; participants frequently cited exposure to material related to emotional intelligence or service recovery training specific to their institution. A summary of participant demographics and performance on the SJT is available in the supplemental appendix.

### Proposed SJT response process model

The study results build on the model proposed by Ployhart (see Fig. 1), which described the SJT response process with four stages: comprehension, retrieval, judgment, and response selection [18]. The new model derived from findings from this study, provided in Fig. 2, includes the four stages as well as additional factors. Factors that are bolded are those with substantial evidence from the cognitive interviews that support their existence (i.e., described in detail in the subsequent sections). The non-bolded factors have limited data to support their inclusion. The proposed model includes all factors identified at least once in the study due to the exploratory purpose; the team decided that even factors with seemingly minor significance could not be excluded due to the small sample size. Within each box connected to the primary stage, factors are arranged by prevalence (i.e., factors higher on the list were referenced more frequently and had a notable presence).

### Comprehension stage

During comprehension, individuals read an item, interpret it, and identify the question [18, 26]. This research identified two features not previously described in the literature: participants often identified a task or objective and participants made assumptions about the scenario. In addition, the comprehension stage includes the ability to identify the construct being assessed [29].

#### Task objective

Participants often identified an objective or task to accomplish in the scenario. Later in the judgment stage, they would evaluate the provided SJT response options based on predictions of how well that response would achieve the objective identified in the comprehension stage. Objectives could often be grouped based on their goals, such as exchanging information, emotional improvement, or problem resolution (Table 2). Of note, many task objectives were broad and lacked a specific focus. For example, participants made general statements about something working well or not without any indication of an explicit goal, such as S15 who said, “that never ends well.”

**Table 2:** Types of task objectives described by participants in the comprehension stage

Task objective type	Description	Example of task objective identification	Example of task objective prediction
Information Exchange	Desire to collect information or share information with another individual	“You want to finish educating thoroughly” (P07)	“You still get the information you need” (S15)
Inconclusive / General	Reference to a non-specific task or objective	“This one was a little difficult in that I didn’t see an end game” (S04)	“Because that never ends well” (S15)
Emotional Improvement	Desire to positively impact feelings or avoid provoking negative feelings	“I was mostly focusing on how to help the patient best to feel better” (S10)	“This can make them more anxious” (S11)
Problem Resolution	Desire to identify or contribute to correcting an issue identified in the item	“I want to identify what can help solve this issue” (S11)	“I think if you do that well, that can really solve the problem” (S05)
Acknowledge	Desire to bring awareness to a challenge or issue	“They want you to validate their sense of loss” (P01)	“They may that you’re just throwing whatever they’ve said under the rug” (P08)
Relationship Modification	Desire to change the interaction between two individuals	“Let them know that they can trust you” (P03)	“That would not establish rapport” (S15)

#### Assumptions

Participants also made assumptions about how they interpreted the case. Assumptions often referred to the person, tone, severity, information accuracy, urgency, or positionality (Table 3). Participants shared assumptions when they believed the scenario lacked sufficient details. P01 best described this by saying, “there’s a fair amount of projection” when interpreting the scenario. Interestingly, SJT scenarios are frequently designed to exclude extraneous information to limit cognitive overload. These data suggest that details about the scenario may be necessary if assumptions in the comprehension process are not desirable.

**Table 3:** Type of assumptions made by participants in the comprehension stage

Assumption types	Description	Example of assumptions
Person	Assumption about the actors within the scenario	“Maybe they are lying but I don’t start with that – I’m not going to assume that” (S04)
Tone	Assumption about how individuals are communicating in the scenario	“It sounded really cold, just you’re required to finish” (S15)
Severity	Assumption about the potential consequences or stakes associated with an outcome of a scenario or response	“Chance are if they got in front of you, it wouldn’t make you late” (S01)
Information Accuracy	Assumption about if the information provided was truthful and complete	“So, if it really was an error … I would first apologize” (P02)
Urgency	Assumption about how quickly the situation needs to be addressed	“I’m going to assume it’s urgent based on that I would apologize” (S04)
Position	Assumption about the relative position of the individual in the scenario	“I’m assuming in the last scenario you’re not on the safety committee” (S04)

#### Ability to identify the construct

Previous research suggests that the examinee’s ability to identify the construct assessed may impact their interpretation and response process [29]. In this study, few participants referenced what they believed the item was measuring—usually, it was statements such as, “I am not sure what I am expected to do here” (P06). Even when asked explicitly during the cognitive interview, participants had difficulty distinguishing empathy consistently.

### Retrieval stage

Retrieval includes selecting knowledge and experiences pertinent to the scenario when formulating a response [18, 26]. For SJTs, the theoretical framework suggests the retrieval stage should promote references to job-specific and general knowledge and experiences [28]. This research also identified that examinees consider their lack of experience or knowledge during their response, which has not been previously described.

#### Job-specific experiences and knowledge

References to job-specific and general experiences (Table 4) often described the location (e.g., the ICU or community pharmacy) and the actors in the scenario (e.g., patients, physicians, nurses). Experiences could also be classified on their similarity to the presented scenario (e.g., how similar or dissimilar to their memory), the specificity of the details provided (e.g., explicit details they recall), and the recency of the experience to the present moment (e.g., within days or weeks). Knowledge references (Table 4) included information, strategies, or skills applied to the scenario, such as legal requirements, direct questions to ask, or broad communication techniques, respectively

**Table 4:** Factors of the experiences and knowledge referenced by participants in the retrieval stage

Factors of experiences and knowledge	Description	Example of experiences and knowledge
*Experience*		
Location	The setting of the experience	“I was called to a different ICU and the patient had an infusion that had been running at the wrong rate” (P11)
Actors	The individuals included in the experience	“I’ve had patients before that have complained to me” (P05)
Task / Topic	The challenge or goal of the experience	“I think anytime you have patients who are upset … you can relate it back to your own experiences” (P06)
Similarity	How consistent their memory is with the presented scenario	“I don’t think I’ve been in a situation very similar to this” (S10)
Specificity	The level of details provided about the experience	“I remember as a resident doing something right, being told by a nephrology resident …” (P10)
Recency	The amount of time between the memory and the experience	“Just actually 2 days ago, the patient we had was on Harvoni …” (P07)
*Knowledge*		
Information	Facts or observations pertinent to the situation	“This one had me immediately thinking about the legal implications of a medication error” (P03)
Strategy	A plan or approach to achieve an objective	“I want to ask them—why they think that, why they want to do that and tell them to talk to their doctor” (S12)
Skill	An ability or set of strategies to achieve an objective	“I just thought about my training … when it comes to our service with hard motivational interviewing” (P14)

#### General experiences and knowledge

General experiences and knowledge (i.e., outside of a healthcare setting) were not referenced often by participants. If discussed, though, references included scenarios about friends or family members in a non-healthcare setting. Notable observations included references to television shows as relevant experiences. For example, when P15 discussed the scenario with a friend taking a medication to help them study, their immediate response included, “Jesse Spano – from Saved by the Bell.” One student, S13, discussed, “I think of experiences that a lot of times I watch on TV shows like Dateline.” General knowledge included references to information such as, “just thinking about social norms, you wouldn’t confront somebody in the grocery store,” as shared by S14. Overall, there was marginal evidence in this study suggesting general experiences and knowledge contributed extensively to SJT response processes.

#### Lack of and nondescript experiences

Participants also included nondescript experiences and references to a lack of experience or knowledge; however, these references were limited. Most participants made statements about broad unfamiliarity with a situation, such as “I don’t really have very much to draw on” (S3) or “this has never happened” (P14). Nondescript examples included instances where P1 stated, “this [question] is a tough one because I feel like this like a reality every day,” and S14 shared, “this one felt familiar to me.”

### Judgment stage

Judgments included utterances about the decision-making process as well as any value statement made while assessing the response options. Factors relevant to this stage included references to emotional intelligence, self-awareness, ability, and impression management [18, 26]. Three new identified factors included: perceptions, feelings about the test, and scenario setting.

#### Emotional intelligence and empathy

One of the most frequent references related to emotional intelligence defined as the capacity to be aware of, control, and express one’s emotions as well as the emotions of others [47]. This was not considered abnormal as the SJT focused on measuring empathy. References to affective and cognitive empathy separately were relatively infrequent; instead, broad references to empathy, such as “putting myself in their shoes” or “this is so sad,” occurred more often and were stated by multiple participants.

#### Self-awareness

Participant commented about themselves in relation to attributes of their personality, their identity, or their comfort with a scenario. For example, individuals shared that the scenario did not resonate with their personality, including comments such as “I think I’m probably a little bit less aggressive” (P11) or “I’m not very confrontational” (S11). References to their identity were typically about their status as healthcare providers, such as P07, who stated, “I guess being a pharmacist, it’s a little clearer.” These references also included identities outside of work. For example, P03 shared that, “as a new parent,” there are differences in how they perceived some situations.

#### Ability

articipants often referenced a lack of skills to complete the tasks instead of affirmations about their ability to succeed. For example, P07 stated that “as a pharmacist, I’m not really trained to walk-through the risks and benefits in that case.” Despite the limited number of ability references, the factor remained in the model as there was some evidence to suggest ability (or the lack thereof) may play a role in response processes. For example, some participants stated they ranked options lower if they did not feel they had the skills necessary to carry it out.

#### Impression management

Participants rarely described that they were intentionally modifying their responses for the person who would review their answers (i.e., impression management) [28]. Most participants reported they forgot to imagine that the test was for selection into a health professions program. The participants who did not forget described a struggle with differentiating their answer choices on what they should do compared to what the individual administering the test would expect them to do. For example, S12 shared they, “kind of knew what the right answer was versus what [they] would actually do was harder to separate.”

#### Perceptions

One new identified factor was that participants shared perceptions that influenced their evaluation of response options (Table 5). For example, participants described how others in the scenario would perceive them if they selected a specific response option. This code is different from impression management, which refers to how the assessor may view the examinee and whether their actions align with the job expectations. Participants often focused on negative impacts such as it could: “make you look like a jerk” (S10), “come off like accusing the patient” (S03), and “seem unprofessional” (P06). Participants also evaluated how the response would be delivered and perceptions about tone. For example, response options that “sounded really cold” (S15) or could “come off a little harsh” (P05) would typically not receive high ratings. Similar to this was the perceived integrity of response options; for example, participants evaluated if the response was an honest reflection of the situation or if the response was legal.

**Table 5:** Types of perceptions described by participants in the judgment stage

Perception types	Description	Examples of perceptions
Image	Perceptions about how the response would reflect on their image as a person	“It just makes you seem lazy” (S03)
They want	Perceptions about what the actor in the scenario would want	“That’s not what they want to hear” (P04)
Integrity	Perceptions about the honesty or legality of a response option	“You’re not portraying the situation how it actually happened” (S10)
Instinct	Perceptions about what inherently feels wrong or right in the scenario	“I did what felt right” (S02)

#### Scenario setting

Another new factor was the role of the item setting—many participants supplemented their selections with “it depends” and other equivalents. Participants cited many factors, such as their role in the scenario, the role of the actors in the scenario, the relationships between themselves and the actors, and historical factors about the scenario. One pharmacist, P06, stated that “If it were a friend, I would have been more inclined to share my own personal experiences … I’d feel more comfortable sharing personal loss and talking about it on a more personal level.” The participant identified that the actor (e.g., friend or patient) and the relationship (e.g., a personal instead of a professional) impacted the response. Participants also explained there are different expectations based on relationships with colleagues compared to patients. For example, one student (S10) shared it is easier to convince a patient (i.e., rather than a friend) not to take a non-prescribed medication “because you could come at it from the standpoint of I’ve had training in this.”

### Response selection stage

The response selection stage included any reference to the final ranking assigned to a response option [18, 26]. Table 6 summarizes the different techniques used by participants in making their final selections.

**Table 6:** Test-taking strategies described by participants in the response selection stage

Test-taking strategies	Description	Example of strategies
*Ordered Approach*		
Best to Worst	Identify responses in order from most to least appropriate	“Going from what would be least conflict inducing to most inducing” (P11)
Worst to Best	Identify responses in order from least to most appropriate	“I started with the least appropriate and worked my way to most” (P04)
Extremes First	Identify responses at the extremes first (least and most appropriate), then the middle	“I identified the first and fifth one” (P06)
Chronologically	Identify responses in order that actions would be performed	“I would do every single one of these in this order” (P10)
Pattern	Identify responses in a type of pattern that is relatively consistent	“I’m noticing a pattern – acknowledge, ask, offer, tell, stay” (S06)
*Compare Responses*	Evaluate response ranking by comparing two at a time	“So, deciding between imagining things and confronting the person” (S12)
*Rephrase*	State the responses in a different way to identify the ranking	“So, what do I do?” (S09)
*Guess*	Randomly assign rankings to a response	“I just kind of put numbers down because I didn’t know” (S12)
*Before Reading Responses*	Attempt to identify the best response before reading the answer options	“Before even looking at the answers, I would think about …” (S02)
*Process of Elimination*	Assign a ranking based on what remains after ranking other options	“I guess through process of elimination it leaves …” (P07)

#### Strategies

Most participants approached the response process in the way they were instructed to, which was to rank responses from most to least appropriate. However, some individuals worked backward (i.e., from least appropriate to most appropriate) in some situations, or they identified the extremes (i.e., most and least appropriate) first and then filled in the remaining ranks. Other strategies included comparing response options, guessing, and using a process of elimination. Some participants, when reading questions aloud, also rephrased the item by orienting themselves within the question. For example, one pharmacist started each response option with “Do you …” when reading the item aloud despite this not being present in the written document.

## Discussion

The use of SJTs in the health professions is a rapidly growing approach to measure professional competence; however, there is a substantial gap in our understanding of the examinee response process when completing an SJT [2]. Current theoretical models by Ployhart suggest the cognitive process during an SJT is similar to models of survey response processes; these include four stages originally described by Tourangeau and colleagues: comprehension, retrieval, judgment, and response selection [18, 26]. Results from this study provided evidence that these stages and several factors are indeed present in SJT response processes according to data from cognitive and think-aloud interviews.

Research outside health professions education previously identified factors that influence SJT response processes, such as job-specific and general experiences, the ability to identify the construct, strategies to select answers, as well as examinee emotional intelligence, self-awareness, ability, and impression management [20, 27, 28]. Results from this study confirmed that job-specific experiences and knowledge, and emotional intelligence were salient factors in the response process. Conversely, factors not sufficiently represented were general experience and knowledge, self-awareness, ability, impression management, and the ability to identify the construct [46, 48]. Our proposed model retains these components as there was not sufficient evidence from this exploratory study to warrant removal. Additional research is necessary to confirm whether to remove these factors. Insufficient evidence for general experiences and knowledge may result from having all study participants with healthcare backgrounds or professional experience to draw on—it is unclear if this would have transpired with participants without healthcare backgrounds [46, 48].

This study also identified new factors involved in response processes that have not previously been described in the literature. For example, findings suggest examinees often attempted to identify a task objective during this SJT, and they evaluate how well response options can achieve that task. In addition, examinees often make assumptions about the scenario that influence how they comprehend and respond to it. We also discovered that examinees sometimes reference nondescript experiences (e.g., television shows), and they also discussed their lack of experiences and knowledge during some SJT items. In the judgment stage, participants also shared that they evaluated response options according to their perceptions about how the action would reflect on their image to others in the scenario. Moreover, participants identified that contextual features such as the item setting could influence their response selections.

Compared to previous research on SJT response processes, this research represents a more in-depth exploration. Moreover, it includes the first investigation of this phenomenon in a health professions context. Rockstuhl and colleagues first reported evidence about SJT response processes; however, they performed the study in a managerial context and only categorized participant utterances during think-aloud interviews about the test content [19]. Our research extended this work by demonstrating how interview data could evaluate the four-stage SJT response process model and elaborate on pertinent factors. Another study conducted by Krumm and colleagues identified some of the strategies test-takers used when completing an SJT [20]. Their research was also focused in a managerial context and was limited in scope; our study identified additional strategies and described the selection process for tests that rank response options.

### Implications and limitations

This is the first study in the health professions to evaluate the salient stages of SJT response processes built from Ployhart’s theoretical model and factors identified in previous research [18, 20, 26, 28, 29]. The research utilized cognitive and think-aloud interviews to evaluate response processes in a step-wise approach, which is considered the standard for provided assessment validity evidence [23, 24]. The results facilitated the generation of an enhanced model to test through future research. The study takes an essential step in generating validity evidence about SJT response processes, which is lacking in SJT research broadly.

Understanding SJT response processes is beneficial because it can inform instrument design in health professions education and the subsequent score interpretation. The study results showcase multiple factors that can contribute to response selection; therefore, SJT design and interpretation should consider these influences. Individuals who use or design SJTs should critically evaluate their SJT items to determine how examinees may interpret the scenario, whether there are sufficient details, and potential assumptions examinees may make that may adversely impact selections. If these factors are believed to influence the response and are not related to the construct of interest, it may be optimal to modify items so that SJT results are more reliable and valid.

A limitation of the presented work is that the relationship between the individual factors in this model and the extent of their influence on response selection is not fully specified. This research explored response processes holistically and evaluated the possible factors present instead of investigating underlying relationships or significance. Future research should consider which components are most influential in SJT performance, how they relate to one another as well as other variables, and whether the factors influence multiple components of the response process rather than a single component as outlined here. Other limitations resulted from methodological choices. For one, this model was constructed using an SJT that measured one construct (i.e., empathy). As such, this model may not apply to other constructs evaluated using an SJT. Future research should test how this model can be applied to SJTs evaluating other constructs.

Furthermore, the study included participants from one profession (i.e., pharmacy) and one region (i.e., southeastern United States). Including students and practicing pharmacists was intentional for a diversity of experiences; however, there may be nuances in a response process model for a novice versus an expert clinician. A larger sample size for statistical comparisons could also identify whether there were unique features to one participant group or question type. Moreover, additional research is necessary to explore whether the model is applicable in other health professions settings (e.g., medicine, nursing) and regions with different experiences or practices.

## Conclusion

The results of this study provide evidence that SJT response processes include four stages as described by Ployhart’s model: comprehension, retrieval, judgment, and response selection. This research used think-aloud and cognitive interviews to describe the factors contributing to response selection and expand the model based on an SJT measuring empathy in a health professions context. The research identified new factors in response processes: identification of tasks or objectives, assumptions about scenarios, perceptions about response options, and the item setting. This study contributes to the literature by expanding the SJT response process model and offers an approach to evaluate SJT response processes further.

## Supplementary Information

**Additional file 1.**

## Data Availability

The datasets generated and/or analyzed during the current study are not publicly available but are available from the corresponding author on reasonable request.

## Abbreviations

SJT: Situational judgment test
P: Pharmacist
S: Student
UNC: University of North Carolina

## References

[1] Koczwara A, Patterson F, Zibarras L, Kerrin M, Irish B, Wilkinson M (2012). Evaluating cognitive ability, knowledge tests, and situational judgment tests for postgraduate selection. Med Educ.

[2] Patterson F, Knight A, Dowell J, Nicholson S, Cousans F, Cleland J (2016). How effective are selection methods in medical education? A systematic review. Med Educ.

[3] Campion MC, Ployhart RE, MacKenzie WI (2014). The state of research on situational judgment tests: a content analysis and directions for future research. Hum Perform.

[4] Chan D, Schmitt N (2002). Situational judgment and job performance. Hum Perform.

[5] Lievens F, Patterson F (2011). The validity and incremental validity of knowledge tests, low-fidelity simulations, and high-fidelity simulations for predicting job performance in advanced-level high-stakes selection. J Appl Psychol.

[6] De Leng WE, Stegers-Jager KM, Husbands A, Dowell JS, Born MP, Themmen APN (2017). Scoring methods of a situational judgment test: influence on internal consistency reliability, adverse impact, and correlation with personality?. Adv Health Sci Educ Theory Pract.

[7] Colbert-Getz JM, Pippitt K, Chan B (2015). Developing a situational judgment test blueprint for assessing the non-cognitive skills of applicants at the University of Utah School of Medicine the United States. J Educ Eval Health Prof.

[8] Patterson F, Ashworth V, Zibarras L, Coan P, Kerrin M, O’Neill P (2012). Evaluations of situational judgement tests to assess non-academic attributes in selection. Med Educ.

[9] Petty-Saphon K, Walker KA, Patterson F, Ashworth V, Edwards H (2016). Situational judgment tests reliably measure professional attributes important for clinical practice. Adv Med Educ Pract.

[10] Wolcott MD, Lupton-Smith C, Cox WC, McLaughlin JE. A 5-minute situational judgment test to assess empathy in first year student pharmacists. Am J Pharm Educ. 2019. 10.5688/ajpe6960. 10.5688/ajpe6960 PMC6718496 31507291

[11] McDaniel MA, List SK, Kepes S (2016). The “hot mess” of situational judgment test construct validity and other issues. Ind Organ Psychol.

[12] Sorrel MA, Olea J, Abad FJ, de la Torre J, Aguado D, Lievens F (2016). Validity and reliability of situational judgment test scores: a new approach based on cognitive diagnosis models. Organ Res Methods.

[13] Caines J, Bridglall BL, Chatterji M (2014). Understanding validity and fairness issues in high-stakes individual testing situations. Qual Assur Educ.

[14] American Educational Research Association, American Psychological Association, National Council on Measurement in Education (2014). Standards for educational and psychological testing.

[15] Fan J, Stuhlman M, Chen L, Weng Q (2016). Both general domain knowledge and situation assessment are needed to better understand how SJTs work. Ind Organ Psychol.

[16] Harris AM, Siedor LE, Fan Y, Listyg B, Carter NT (2016). In defense of the situation: an interactionist explanation for performance on situational judgment tests. Ind Organ Psychol.

[17] Melchers KG, Kleinmann M (2016). Why situational judgment is a missing component in the theory of SJTs. Ind Organ Psychol.

[18] Ployhart RE, Weekly JA, Ployhart RE (2006). The predictor response process model. Situational judgement tests: theory, measurement, and application.

[19] Rockstuhl T, Ang S, Ng KY, Lievens F, Van Dyne L (2015). Putting judging situations into situational judgment tests: evidence from intercultural multimedia SJTs. J Appl Psychol.

[20] Krumm S, Lievens F, Huffmeier J, Lipnevich AA, Bendels H, Hertel G (2015). How “situational” is judgment in situational judgment tests?. J Appl Psychol.

[21] Pellegrino J, Chudowsky N, Glaser R (2001). Knowing what students know: the science and design of educational assessment. Board on Testing and Assessment, Center for Education, National Research Council, Division of Behavioral and Social Sciences and Education.

[22] Nichols P, Huff K, Ercikan K, Pellegrino JW (2017). Assessments of complex thinking. Validation of score meaning for the next generation of assessments: the use of response processes.

[23] Leighton JP (2017). Using think-aloud interviews and cognitive labs in educational research: understanding qualitative research.

[24] Willis GB (2015). Analysis of the cognitive interview in questionnaire design: understanding qualitative research.

[25] Schwarz N (2007). Cognitive aspects of survey methodology. Appl Cogn Psychol.

[26] Tourangeau R, Rips LC, Rasinski K (2000). The psychology of survey response.

[27] Brooks ME, Highhouse S, Weekly JA, Ployhart RE (2006). Can good judgment be measured?. Situational judgement tests: theory, measurement, and application.

[28] Lievens F, Motowidlo SJ (2016). Situational judgment tests: from measures of situational judgment to measures of general domain knowledge. Ind Organ Psychol.

[29] Griffin B (2014). The ability to identify criteria: its relationship with social understanding, preparation, and impression management in affecting predictor performance in a high-stakes selection context. Hum Perform.

[30] Kim SS, Kaplowitz S, Johnston MV (2004). The effects of physician empathy on patient satisfaction and compliance. Eval Health Prof.

[31] Riess H (2017). The science of empathy. J Patient Exp.

[32] Decety J, Jackson PI (2004). The functional architecture of human empathy. Behav Cogn Neurosci Rev.

[33] Hojat M (2007). Empathy in patient care: antecedents, development, measurement, and outcomes.

[34] Quince T, Thiemann P, Benson J, Hyde S (2016). Undergraduate medical students’ empathy: current perspectives. Adv Med Educ Pract.

[35] Tamayo CA, Rizkalla MN, Henderson KK (2015). Cognitive, behavioral, and emotional empathy in pharmacy students: Targeting programs for curriculum modification. Front Pharmacol.

[36] Fjortoft N, Van Winkle LJ, Hojat M (2011). Measuring empathy in pharmacy students. Am J Pharm Educ.

[37] Nunes P, Williams S, Sa B, Stevenson K (2011). A study of empathy decline in students from five health disciplines during their first year of training. Int J Med Educ.

[38] Lievens F (2017). Construct-driven SJTs: toward an agenda for future research. Int J Test.

[39] McDaniel MA, Hartman NS, Whetzel D, Grubb WL (2007). Situational judgment tests, response instructions, and validity: a meta-analysis. Pers Psychol.

[40] McDaniel MA, Nguyen NT (2001). Situational judgment tests: a review of practice and constructs assessed. Int J Sel Assess.

[41] Patterson F, Zibarras L, Ashworth V (2016). Situational judgement tests in medical education and training: research, theory, and practice: AMEE guide no. 100. Med Teach.

[42] Weekley JA, Ployhart RE, Weekly JA, Ployhart RE (2006). An introduction to situational judgment testing. Situational judgement tests: theory, measurement, and application.

[43] Wolcott MD, Lobczowski NG. Using cognitive interviews and think-aloud protocols to understand thought processes in education research. Curr in Pharm Teach Learn. 2020. In press. 10.1016/j.cptl.2020.09.005. Epub 14 Oct 2020. 10.1016/j.cptl.2020.09.005 33454077

[44] Saldana J (2016). The coding manual for qualitative researchers.

[45] Merriam S, Tisdell EJ. Qualitative research: a guide to design and implementation. San Francisco: Jossey-Bass; 2016.

[46] Wolcott MD. The situational judgment test validity void: Describing participant response processes [doctoral dissertation]: University of North Carolina; 2019. Retrieved from ProQuest Dissertations & Theses., Accession Number 10981238.

[47] Cherry MG, Fletcher I, O’Sullivan H, Dornan T. Emotional intelligence in medical education: a critical review. Med Educ. 2014;48(5):468–78. 10.1111/medu.12406 24712932

[48] Wolcott MD, Lobczowski NG, Zeeman JM, McLaughlin JE. Exploring the role of knowledge and experience in responses to situational judgment test responses using mixed methods. Am J Pharm Educ. [in press] https://doi.org/10.5688/ajpe8194. Epub Sep 2020.

