# Supplementary material for: Situational judgment test validity: an exploratory model of the participant response process using cognitive and think-aloud interviews
Source: BMC Med Educ. 2020 Dec 14;20:506. doi: 10.1186/s12909-020-02410-z (PMC7734708; doi:10.1186/s12909-020-02410-z)
Supplement: Supplementary file 1 — Additional file 1. [file 12909_2020_2410_MOESM1_ESM.docx]

# Supplemental Appendix 1. Map of SJT Items


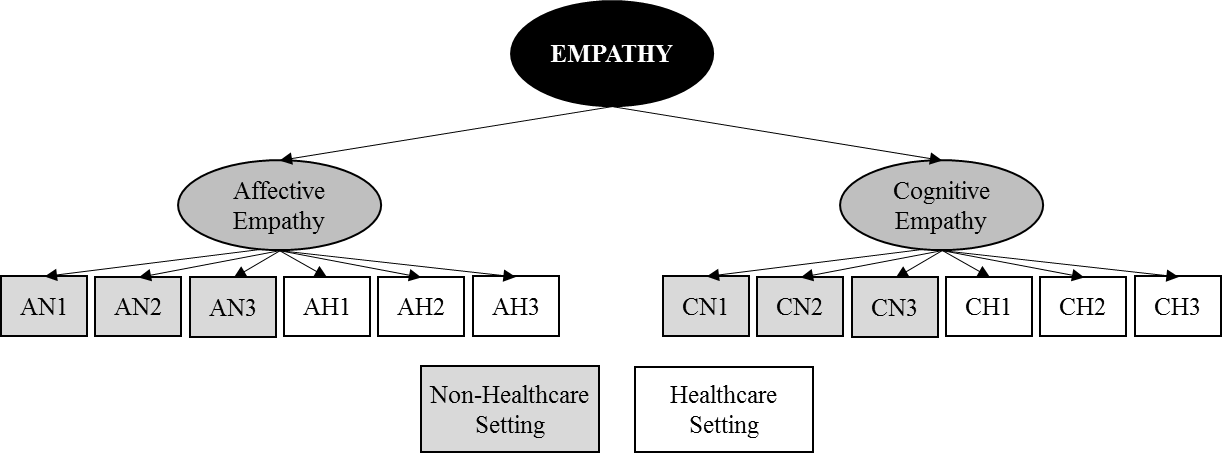


*Figure*: Map of SJT items, settings, and the associated construct components. A = Affective Empathy; C = Cognitive Empathy; H = Healthcare Setting, N = Non-Healthcare Setting; 1, 2, 3 = Item Number

# Supplemental Appendix 2. SJT Response Process Final Codebook

| Code (Abbv) | Description | Samples |
| --- | --- | --- |
| Comprehension (CO) | The cognitive process used by the examinee to read, interpret, or understand the purpose of the test item. | “The way I interpret this”, “This sounds like”, “I didn’t read carefully” |
| Task Objective (OB)* | Identification or prediction of a goal to be accomplished. | “What would be best for the patient”, “The patient may take that in a bad way” |
| Assumptions (AM)* | Interpretations or constraints placed on the scenario based on the perspective of the examinee. | “I assume this is said in a polite tone”, “I think this comes off as…”, “I am assuming there is…” |
| Ability To Identify Construct (AC) | The examinee’s attempt to identify which attribute is being evaluated by a test question. | “I think this is asking me to”, “I’m not sure what I am expected to do here” |
| Retrieval (RT) | Accessing long-term memories and knowledge relevant to the scenario and proposed problem. | “This makes me think of…”, “I remember…” |
| Specific Job Experience (JE) | Memories or observations that are related to experiences exclusively within the health professions. | “I remember a time in the hospital”, “I work with patients who have depression every day” |
| Specific Job Knowledge (JK) | Facts, information, strategies, or skills identified to address problems that are encountered exclusively within the health professions. | “We are trained in mental health first aid”, “We have a policy that”, “It depends how they manage their diabetes” |
| General Experience (GE) | Memories or observations that are related to experiences outside of the health professions. | “I’ve had friends who have gone through loss”, “Reminds me when I would vent to a friend” |
| General Knowledge (GK) | Facts, information, strategies, or skills identified to address problems that are encountered in contexts outside of the health professions and broadly applicable to societal or cultural expectations. | “I think there’s social norms still… you’re not going to let them start a fist fight in the grocery store”, “We have university policies” |
| Lack Experience (LE)* | Reference to not having witnessed or encountered a scenario or setting that is described. | “I can’t think of a time…”, “I have not seen this before…” |
| Nondescript Experience or Knowledge (NE)* | Memories, observations, facts, information, strategies, or skills provided without a clear distinction of the setting or environment in which they occurred. | “This has happened to me” (with no qualifiers to distinguish the setting), “This happens all the time” |
| Judgment (JU) | Making a decision or value-statement; typically generated by integrating memories, knowledge, experiences, and other antecedents. | “This is a bad approach”, “I think that is a good idea”, “I would never do that”, “Compared to this option”, “You should...”. NOT: “Yes”, “No” |
| Emotional Intelligence (EI) | The capacity to be aware of, control, and express one’s emotions and to handle relationships. | “They want you to validate their feelings”, “That is upsetting” |
| Affective Empathy (AE) | Individual’s ability to experience and internalize the feelings experienced by others. | “They are likely upset or frustrated”, “This is so sad” |
| Cognitive Empathy (CE) | Individual’s ability to understand another person’s perspective instead of being self-oriented. | “Trying to think about their perspective”, “Putting myself in their shoes…” |
| Ability (AB) | Reference to the possession or lack of the means or skills to do something such as a talent, skill, or proficiency in a particular area. | “I don’t know how to do that well”, “I’m not really trained to…”, “If I was more skilled at…” |
| Self-Awareness (SA) | Awareness of the characteristics or qualities that form an individual’s character or identity. | “As a pharmacist…”, “It makes me uncomfortable”, “I tend to be more judgy” |
| Impression Management (IM) | Extent to which an examinee modifies a response based on what is expected from the test administrator or assessor. | “The residency program director would want me to”, “I’d want to look like I am compassionate” |
| Perceptions (PR)* | Awareness of what others in the test scenario may think about the examinee. | “It just makes you seem lazy” |
| Context (CT)* | Reference to how variations in the components of the scenario may affect the selected responses. | “It depends on…”, “I don’t think my answer would change in a healthcare setting” |
| Response Selection (RP) | The final verbal or written answer that is selected by the examinee. | “This would be number five”, “It goes last” |
| Strategies (ST) | Techniques used by examinees to answer test items. | “I selected the first and last option, then guessed” |

*****Code added through inductive process (i.e., not in the original codebook)

# Supplemental Appendix 3. Think-Aloud Interview Script

*The following script was adopted from Leighton.^23^*

Thank you for attending the session today.

Today’s session will be divided into two parts. In the first part, you will complete twelve (12) questions on a fictitious exam that could be used to evaluate potential residents for a residency program or students for a health professions program. For each question, you will be given a scenario and requested to rank the response options based on how you *should* respond to the scenario. Your rankings should be labeled 1 for the most appropriate and 5 for the least appropriate with no ties or duplicates. In the second part, I will be asking you specific questions about a randomly selected set of eight (8) questions.

For the first part of this study, I am interested in learning about the thoughts you have as you answer. For this reason, I am going to ask you to think aloud as you work through the test. Let me explain what I mean by “think aloud”. It means that I would like you to tell me everything you think about as you work through each test question. You will do this one test question at a time.

When I say tell me everything, I really mean every thought you have from the moment you read the problem to the end when you have a solution or even if you do not have a solution. Please do not worry about planning how to say things or clarifying your thoughts. What I really want is to hear your thoughts constantly as you try to solve the problem – uninterrupted and unedited. Sometimes you may need time to think quietly through something – if so, this is okay but please tell me what you thought through as soon as possible after you are finished.

I realize it can feel awkward to think aloud but try to imagine you are alone in the room. If you become silent for too long, I will say “keep talking” to remind you to think aloud. Please note, this research is highly exploratory. My intention is not to evaluate your thinking or explanations while you speak. The purpose of the study is to learn about the thoughts as you—and other people—answer each question.

We will have an opportunity to practice, but before we get to that, please let me know if you understand what we will be doing today.

Do you have any questions?

Let us now practice thinking aloud with two practice problems presented on your paper.

- Lucas works 7.5 hours in a day. How many hours does he work in 5 days? *Now, please tell me everything that you are thinking as you try to solve this*.
- What is the 5^th^ letter after C? *Now, please tell me everything that you are thinking as you try to solve this*.

# Supplemental Appendix 4. Cognitive Interview Script

*The following script was adopted from Leighton.^23^*

*Begin the interview and start with the first selected test question – after the participant reviews each question, the interviewer will ask the following* *if it was not addressed by the participant:*

For this next part, I will ask you a series of questions about each question – they will become repetitive. Please be succinct in your responses. At the end, I will ask some general questions about the test as a whole.

Do you have any questions?

- How did you decide how to rank each option?
  - *Further probe:* What made your decisions easier and why?
  - *Further probe:* What made your decisions harder and why?
- What, if any, experiences does this question make you think of when you answered the question?
  - *Further probe:* What memories did you think about when you answered the question?
- What if the setting of this question was different, how does that impact your response?
  - *Further probe*: What rank would you have assigned each response if the question had been in a setting that was (non-)healthcare-related?
  - *Further probe*: Was there wording about this question that influenced your response?
- What knowledge or ability do you think this question is testing? Why do you think this?
  - *Alternative phrasing:* What do you think this question is asking you to do and why?

*The interview may conclude with the following questions based on time*:

- What questions do you feel were easiest to answer and why?
  - What questions do you feel were difficult to answer and why?
  - How did ranking each option influence your response?
- In general, what factors do you believe influenced your response to each scenario?
- If you had known all of these questions were testing empathy, how would that have changed your responses?
- How did knowing that this test may be used for residency selection influence your responses?
- What questions made you feel confused and why? Do you feel you did not understand some of the questions?
- How would your responses have differed if the questions were open-ended?

The last part of this session includes a brief 5-minute questionnaire. Once you have finished the questionnaire the session is complete. Thank you again for your participation.

# Supplemental Appendix 5. SJT Study Participant Characteristics Table (N = 30)

|  | Students  (n = 15) | Pharmacists  (n = 15) |
| --- | --- | --- |
| Male, n (%) | 4 (27) | 2 (13) |
| Age | 24 (22-45) | 36 (29–51) |
| Anticipated graduation year, n (%) |  |  |
| Class of 2019 | 4 (27) | *** |
| Class of 2020 | 7 (40) | *** |
| Class of 2021 | 4 (27) | *** |
| Education and training, n (%) |  |  |
| Bachelor of Science Degree | 15 (100) | *** |
| Doctor of Pharmacy | *** | 15 (100) |
| Residency (e.g. PGY1 and/or PGY2) | *** | 13 (87) |
| Fellowship or post-doc | *** | 1 (7) |
| Advanced degree (e.g. MPH, MBA, PhD) | *** | 3 (20) |
| Board certification (e.g. BCPS, BCOP) | *** | 11 (73) |
| Practice Location, n (%) |  |  |
| University hospital A | *** | 11 (73) |
| University hospital B | *** | 4 (27) |
| Practice area, n (%) |  |  |
| Academia | *** | 2 (13) |
| Administration | *** | 1 (7) |
| Ambulatory care | *** | 1 (7) |
| Cardiology / pulmonology | *** | 2 (13) |
| Critical care / emergency medicine | *** | 3 (20) |
| General medicine | *** | 2 (13) |
| Infectious diseases | *** | 2 (13) |
| Psychiatry | *** | 1 (7) |
| Surgery | *** | 1 (7) |
| Work experience in a healthcare-related field, n (%) | 13 (87) | 15 (100) |
| Years licensed as a pharmacist | *** | 8 (6–23) |
| Years with a health professions faculty appointment | *** | 5 (0–20) |
| Average number of hours working in a healthcare setting per week | 5 (0–40) | 40 (0–55) |
| Average number of patients interacting with per week | 3 (0–75) | 20 (0–100) |
| Average number of students interacting with per week | *** | 2 (0–8) |
| Average number of non-pharmacist healthcare providers interacting with per week | 2 (0–10) | 10 (2–35) |
| Work experience in a nonhealthcare-related human services field, n (%) | 8 (53) | 11 (73) |
| Years of work experience in a nonhealthcare-related human services field | 1 (0–10) | 4 (0–10) |
| Experience taking care of a terminally ill family member or individual, n (%) | 1 (7) | 4 (27) |
| Questionnaire of Cognitive and Affective Empathy (QCAE) Score | 93 (79–103) | 90 (85–105) |
| Cognitive Empathy (CE) Score | 57 (46–67) | 58 (50–68) |
| Affective Empathy (AE) Score | 37 (27–42) | 34 (29–39) |
| Self-reported training related to empathy, n (%) | 12 (80) | 5 (33) |

# Supplemental Appendix 6. SJT Item Psychometrics Based on All Responses (N = 30)

|  | *M* | *SD* | *r* | *Min* | *Max* | *W* |
| --- | --- | --- | --- | --- | --- | --- |
| CH1 | 15.6 | 3.1 | .23 | 10 | 20 | .66 |
| CH2 | 15.1 | 2.7 | .06 | 12 | 20 | .65 |
| CH3 | 17.1 | 2.5 | .14 | 12 | 20 | .81 |
| CN1 | 13.8 | 2.8 | .26 | 8 | 20 | .76 |
| CN2 | 15.0 | 2.6 | .63 | 10 | 18 | .54 |
| CN3 | 13.9 | 3.3 | .38 | 8 | 20 | .68 |
| AH1 | 15.7 | 3.6 | .49 | 8 | 20 | .69 |
| AH2 | 13.2 | 3.0 | .30 | 8 | 20 | .56 |
| AH3 | 17.3 | 1.9 | .38 | 14 | 20 | .85 |
| AN1 | 14.7 | 3.4 | .40 | 8 | 20 | .77 |
| AN2 | 15.5 | 2.6 | .35 | 10 | 20 | .66 |
| AN3 | 13.7 | 3.0 | .08 | 8 | 20 | .39 |
| *TOTAL TEST* | 180.6 | 11.8 | *** | 142 | 200 | *** |

Notes: M = mean, SD = standard deviation, r = discrimination index (Pearson’s r),

Min = minimum score, Max = maximum, score, W = Kendall’s coefficient of concordance
